# Four-year longitudinal associations of physical activity, waist circumference, and blood pressure in UK adolescents

**DOI:** 10.1038/s41390-023-02837-2

**Published:** 2023-10-13

**Authors:** Sally P. Waterworth, Catherine J. Kerr, Christopher J. McManus, Henry C. Chung, Brandon S. Shaw, Ina Shaw, Gavin R. Sandercock

**Affiliations:** School of Sport, Rehabilitation and Exercise Sciences, University of Essex, Wivenhoe Park, Colchester, Essex CO4 3SQ UK

## Abstract

**Background:**

This study assessed the specific influence of physical activity (PA) and waist circumference (WC) on the 4-year growth trajectory of blood pressure in UK high-school students.

**Methods:**

Four-year longitudinal monitoring of 1501 adolescents was conducted as part of the EoEHHS. Measurements were taken in Grades (G)7, 9, and 11.

**Results:**

Systolic (SBP) and diastolic blood pressure (DBP) increased over the 4-year period (SBP G7 114.6 ± 8.9 mmHg, G9 118.1 ± 9.7 mmHg, G11 122.8 ± 7.8 mmHg; DBP G7 66.7 ± 6.6 mmHg, G9 68.0 ± 6.4 mmHg, G11 70.0 ± 5.2 mmHg). Baseline WC predicted baseline and growth in SBP, but the strongest contribution to SBP came from changes in WC (*β* = 0.084, *p* = 0.002). Baseline PAQ-A score (*β* = −0.822, *p* = 0.020) and changes in PAQ-A score (*β* = −0.650, *p* = 0.019) were associated with smaller increases in DBP over the 4-year measurement period.

**Conclusions:**

Baseline and change in WC predicted the growth trajectory of SBP, while baseline and change in PA predicted the growth trajectory of DBP. PA and WC have a prognostic value in predicting changes in blood pressure in adolescents. Increasing PA during adolescence could slow the rise in DBP over time. This is meaningful for future hypertension and CVD risk reduction into adulthood.

**Impact:**

- Hypertension in adolescents is a growing health problem that is often overlooked.
- Baseline and changes in waist circumference over a 4-year period predicted development of systolic blood pressure, while baseline and changes in physical activity predicted development of diastolic blood pressure.
- Physical activity and waist circumference have a prognostic value in predicting changes in blood pressure in adolescents and could be valuable in planning programmes to prevent hypertension in similar communities and reduce the risk of future adult hypertension.

## Introduction

Hypertension is a major contributor to cardiovascular disease (CVD) and early mortality among adults.^1^ The United Kingdom (UK) reference data for blood pressure centiles demonstrate that systolic blood pressure (SBP) and diastolic blood pressure (DBP) increase with age in children and adolescents. The rise is consistent with the influence of body mass and stature on blood pressure (BP).^2^ There is a tendency for individuals to maintain the same BP rank relative to their peer group over time, referred to as tracking. Higher childhood BPs tend to track into adulthood,^3,4^ where they are associated with pathologic changes that include atherosclerotic development and altered cardiac morphology.^5^ A meta-regression analysis reported mean tracking coefficients of 0.38 for SBP and 0.28 for DBP.^3^ These, however, vary with the age of the cohort and the length of follow up and ranges between −0.12 and 0.80 for SBP and between −0.16 and 0.70 for DBP have been reported.^3^ These tracking coefficients are moderate, suggesting that BP is modifiable. Identifying factors associated with longitudinal changes/trajectories in BP is therefore important to prevent elevated BP in childhood progressing to adult hypertension.

Adiposity and physical activity (PA) are modifiable risk factors for elevated BP in childhood, and later adulthood.^6^ Specifically, waist circumference (WC), as a measure of central adiposity, is positively and independently associated with youth BP (*β* = 0.094–0.260).^7^ Further, prospective studies have reported positive correlations between adolescent WC and adult BP.^8^ The health benefits of PA are well established, and higher PA levels in adolescents generally have a negative relationship with increased BP,^9–11^ and thus are protective against the development of hypertension.^12^ The positive effects of PA on BP are independent of changes in weight status and obesity.^13^ Typically, longitudinal studies use regression analysis to identify baseline characteristics that predict follow-up values or changes in BP. This approach, however, cannot account for the combined effects of initial levels and changes in correlates of BP, such as PA and adiposity, both of which change during adolescence and differ by sex. There are no longitudinal data available on changes in blood pressure in English youth, and no studies assessing the impact of PA and adiposity on development of BP. Understanding the progression of BP in children may prove an important preventative measure in public health due to the relationship between increases in childhood BP and an increased risk of hypertension in adulthood. The aim of this study was to use latent growth curve modelling (LGM) to assess the effects of initial values and longitudinal changes in PA and WC adiposity on the growth trajectory of BP in UK high-school students.

## Methods

### East of England Healthy Hearts Study

As described elsewhere in detail,^14^ the East of England Healthy Hearts Study (EoEHHS) 2006–2011 was a regional and school-based study. The study sample of 1501 Grade 7 scholars from 23 schools in the south-east region of England volunteered to participate in the longitudinal monitoring study (season-matched follow-up assessments were replicated after 2 (Grade 9) and 4 years (Grade 11). Parents gave written informed consent before data collection commenced. Ethical approval was granted by the researchers’ institutional review board and conforms with the tenets of the Declaration of Helsinki as revised in 2013.

### Protocol

#### Geographic entities

The English Indices of Multiple Deprivation (IMD) 2007 was measured based on the small-area geographical units known as lower super output areas (LSOAs), each LSOA containing 1000–3000 inhabitants.^15,16^ The level of deprivation within each of the 32,482 LSOAs in England is represented by a multicomponent score with values ranging from 0.4 (least deprived) to 85.5 (most deprived).

#### Anthropometry

For descriptive purposes, body weight was measured to the nearest 0.1 kg using a digital flat scale (Seca 875, Hamburg) and stature was measured to the nearest 0.1 cm using a portable stadiometer (Seca 213, Hamburg). Waist circumference (WC) was taken 2 cm above the umbilicus by means of a non-distendable measuring tape (Bodycare Products, Southam, UK) to the nearest 1 mm. Repeat measures were taken at a similar time to initial measures and the same equipment was used throughout.

#### Physical activity levels

Each participant completed the Physical Activity Questionnaire for Adolescents (PAQ-A), which is a self-administered 7-day recall instrument. The questionnaire design and administration have been described in detail elsewhere.^17^ Briefly, the questionnaire comprised three sections including overall PA, activity within school, and activities undertaken in the evenings and at weekends. Total PAQ-A scores were calculated and combined with individual components of the questionnaire to form continuous measures which were used in subsequent analyses.^18^ The North-American version of PAQ-A was anglicised (e.g. recess became break; soccer became football); a full description of these changes, application, scoring, validity and reliability of the PAQ-A have been reported previously.^19^

#### Blood pressure

Blood pressure was assessed prior to any physical exertion, generally after participants completed the basic study self-reported information and PAQ-A (i.e. after 5–10 min of sitting). Measurements were made after a further 5 min of quiet, seated rest using an automated oscillometric sphygmomanometer (Omron MX3, Omron Healthcare Europe BV, Hoofddorp, The Netherlands). Researchers fitted an appropriately sized inflatable cuff (child cuff 10–19 cm, small adult cuff 17–25 cm) around the upper left arm of each participant. Participants were instructed to sit still in their chair with feet flat on the floor, and legs uncrossed and left forearm resting on a table with the palm facing upwards. A minimum of two measurements were obtained for both systolic (SBP) and diastolic (DBP) blood pressure and were recorded in millimetres mercury (mmHg). The lowest values were recorded as the first reading in a series of BP measurements, as these are typically higher when using an oscillometric measurement device.^20^ In the case of measurement error or when there was poor agreement between measures (difference of >20 mmHg SBP or >10 mmHg DBP), a third measurement was made.

#### Statistical analyses

**Fig. 1:**
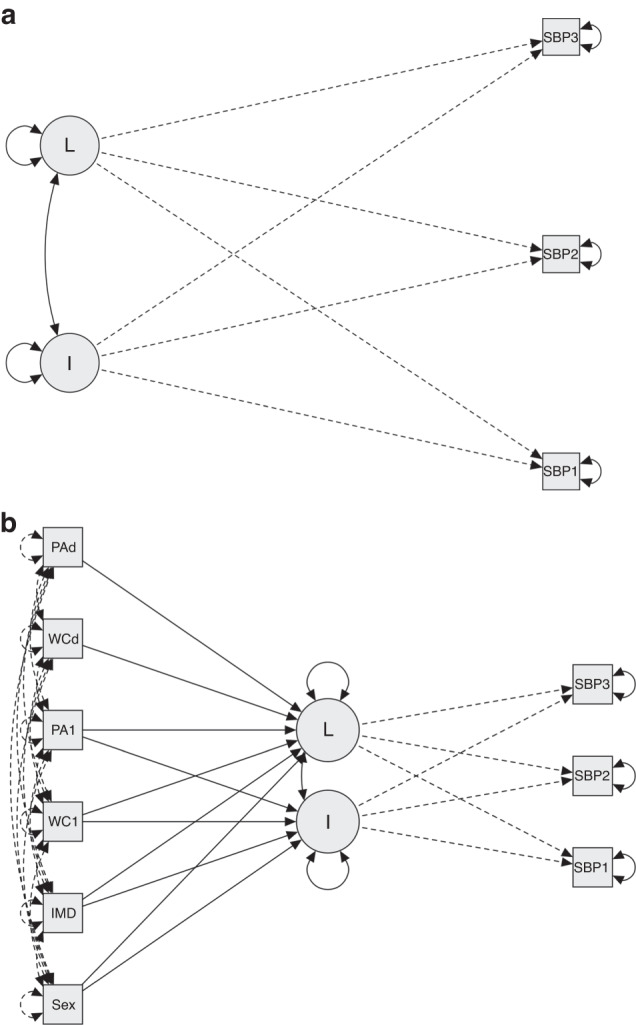
Path diagrams for latent growth curve models. Path diagrams for **a** basic latent growth curve model and **b** adjusted latent growth curve model. *I* and *L* are both latent variables derived from the observed variables for (in this case) systolic blood pressure (SBP) measured at three time points. Baseline measures affect both the intercept and slope of the growth models, whereas change in waist circumference and physical activity only affect the slope of the growth models. I = intercept, L = latent growth curve slope; SBP 1 = Grade 7; SBP 2 = Grade 9; SBP 3 = Grade 11; S.1 = sex; IMD = Index of Multiple Deprivation; PA = physical activity, WC = waist circumference, PAd. = difference (change) in physical activity over 4 years; WCd. = -difference (change) in waist circumference over 4 years.

Latent growth curve modelling (LGM) was used to study the development in SBP and DBP over the three assessment points (Year (Y)0, Y2 and Y4 at Grades 7, 9 and 11, respectively). LGM is a statistical approach which allows the simultaneous modelling of initial values and change in values and has the benefit of being able to examine both differences between individuals and changes within individuals (Fig. 1).^21^ A unique feature of LGM is that it considers both the means and covariances of repeatedly measured variables in the analysis, allowing statistical examination of the hypothesised means of variables and covariances among variables at the same time.^22^ Supplementary Information 1 provides complete details on the modelling procedures including the initial goodness-of-fit statistics for each unrestrained latent growth model curve and the unstandardised estimates for each model. Unconditional linear growth models were first fitted without additional predictors for SBP and DBP (Fig. 1a). The intercept and slope (growth parameters) were specified as latent variables. Restraining the intercept to a value of 1 represents the initial level of BP and the slope represents the rate of change in BP over time. Changes in BP over time were initially assumed to be linear and this was tested assessing the goodness-of-fit statistics for the covarying linear model in each case. This illustrates how the three endogenous variables (BP at Y0 Y2 Y4) were predictors of both the two latent variables of intercept and slope, which were allowed to covary in the model.

Maximum likelihood estimation with the full information maximum likelihood algorithm (FIML) was used to account for missing values. FIML provides a robust index of fit when data are missing at random, the initial sample size is sufficiently large (*n* = 1501) and when endogenous variables are normally distributed.

To evaluate the goodness-of-fit of the initial models, the *χ*^2^ test, Comparative Fit Index (CFI), the Tucker–Lewis Index (TLI), the Root Mean Square Error of Approximation (RMSEA), and the Standardised Root-Mean-Square Residual (SRMR) were used (Supplementary Information 1). The model fits the data well if the *p* value for the *χ*^2^ test is non-significant, CFI and TLI values are close to 0.95, the RMSEA value is below 0.06, and the SRMR value is below 0.08.^23^

After confirming the satisfactory fit for each unconditional model, baseline (Y0, Grade 7) values for deprivation (IMD), WC and PAQ-A score were introduced as predictors of the initial level (intercept) and development (slope) of BP (Fig. 1b).

To assess the impact that change in predictor variables had on BP over the four-year period, change values (Δ) were calculated for WC and PAQ-A score. For each predictor, Δ-values were obtained by subtracting initial (i.e. Y0) values from Y4 values. These Δ-values were included in regression analyses as predictors of the latent variable level (slope) to determine their influence on change in SBP and DPB over time. The regression coefficients for the predictor variables represent 1-year changes in WC and PAQ-A score.

## Results

Descriptive characteristics of participants are shown in Table 1. According to 91st and 98th centile cut-offs,^2^ SBP was normotensive in 80.5% of boys and 84.9% of girls, pre-hypertensive in 12.3% of boys and 9.3% of girls, and hypertensive in 7.2% of boys and 5.9% of girls. DBP was normotensive in 63.4% of boys and 58.7% of girls, pre-hypertensive in 21.3% of boys and 22% of girls, and hypertensive in 15.3% of boys and 19.3% of girls. According to 91^st^ and 98^th^ centile cut-offs for WC, 68.5% of boys and 68.6% of girls were normal weight, 12.5% of boys and 9.7% of girls were overweight, and 19.1% of boys and 21.7% of girls were obese.

**Table 1:** Descriptive characteristics of participants.

		Males			Females			All		
		Grade 7	Grade 9	Grade 11	Grade 7	Grade 9	Grade 11	Grade 7	Grade 9	Grade 11
Complete cases		*N* = 772	*N* = 760	*N* = 499	*N* = 650	*N* = 662	*N* = 417	*N* = 1422	*N* = 1422	*N* = 916
Deprivation (IMD)	Mean (SD)	11.6 (7.3)	11.7 (7.3)	11.3 (7.2)	11.4 (6.9)	11.3 (6.9)	11.2 (6.8)	11.5 (7.1)	11.5 (7.1)	11.3 (7.0)
Age (years)	Mean (SD)	12.0 (.5)	14.0 (.4)	15.9 (.4)	12.0 (.4)	14.0 (.4)	15.9 (.4)	12.0 (.4)	14.0 (.4)	15.9 (.4)
Stature (cm)	Mean (SD)	151.1 (7.7)	163.0 (8.1)	166.4 (7.7)	152.2 (7.2)	161.8 (7.8)	165.1 (7.6)	151.6 (7.5)	162.4 (8.0)	165.8 (7.6)
Body mass (kg)	Mean (SD)	43.9 (10.0)	53.9 (9.1)	57.1 (7.1)	45.4 (9.4)	53.4 (9.1)	56.4 (6.9)	44.6 (9.8)	53.7 (9.1)	56.8 (7.0)
BMI (kg/m^2^)	Mean (SD)	19.1 (3.5)	20.3 (2.9)	20.6 (2.3)	19.5 (3.2)	20.4 (3.0)	20.7 (2.2)	19.3 (3.4)	20.3 (3.0)	20.7 (2.3)
Waist (cm)	Mean (SD)	66.8 (8.0)	70.1 (6.9)	71.8 (5.5)	64.8 (8.1)	68.9 (6.9)	71.4 (5.4)	65.9 (8.1)	69.5 (6.9)	71.6 (5.5)
Physical activity (PAQ)	Mean (SD)	3.0 (0.5)	2.7 (0.5)	2.6 (0.5)	2.7 (0.5)	2.6 (0.5)	2.4 (0.4)	2.9 (0.5)	2.7 (0.5)	2.5 (0.5)
Systolic BP (mmHg)	Mean (SD)	114.5 (8.5)	118.3 (9.8)	123.3 (7.9)	114.6 (9.4)	117.9 (9.7)	122.3 (7.6)	114.6 (8.9)	118.1 (9.7)	122.8 (7.8)
Diastolic BP (mmHg)	Mean (SD)	66.1 (6.4)	68.0 (6.3)	69.9 (5.4)	67.4 (6.9)	68.0 (6.6)	70.0 (5.0)	66.7 (6.6)	68.0 (6.4)	70.0 (5.2)

### Missing data

The frequency and patterns of missing data points for all independent and dependent variables across the three measurement points are shown in Supplementary Information 2. Complete data were available for 73.1% (*n* = 1039) of participants at all three measurement points. Overall, there were *n* = 473 missing values for BP, WC, and physical activity (PA)—equivalent to 2.7% of all values potentially available across the three measurement points.

Little’s MCAR test was not significant (*χ*^2^ = 627.3, df = 572, *p* = 0.054) indicating that values were missing completely at random. Importantly, there were no meaningful differences in measures of BP, WC, or PA (all *d* < 0.2). Based on this evidence, data were confirmed as missing completely at random (missingness could not be accounted for by the values of variables themselves nor explained by the values of other variables included in the analysis).

### Trends in outcomes

**Fig. 2:**
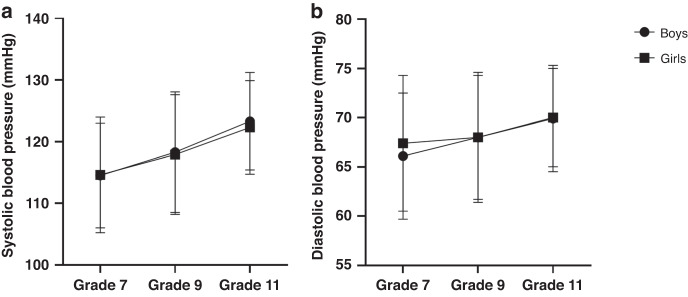
Blood pressure measurements across a 4-year period. Mean (±SD) **a** systolic and **b** diastolic blood pressure measurements across a 4-year period for boys and girls. Both systolic (SBP) and diastolic blood pressure (DBP) increased during the 4-year observational period in boys and girls. In boys, SBP increased from 114.5 ± 8.5 mm mercury (mmHg) at Grade 7 to 123.3 ± 7.9 mmHg at Grade 11 and DBP from 66.1 ± 6.4 mmHg at Grade 7 to 69.9 ± 5.4 mmHg at Grade 11. In girls, SBP increased from 114.6 ± 9.4 mmHg at Grade 7 to 122.3 ± 7.6 mmHg at Grade 11 and DBP from 67.4 ± 6.4 mmHg at Grade 7 to 70.0 ± 5.0 mmHg at Grade 11.

Unstandardised mean estimates and variance in intercept and slope for SBP and DBP are shown in Table 2. Latent covariances between intercept and slope showed a significant negative association for SBP (*β* = −3.57 [1.36], *p* = 0.09) and for DBP (*β* = −1.45 [0.74], *p* = 0.04). The mean values for SBP and DBP are presented in Fig. 2. Both SBP and DBP increased during the 4-year observational period in boys and girls. Model fit parameters showed that the trend was linear (Supplementary Information 1).

**Table 2:** Unstandardised mean estimates and variance in intercept and slope.

	Intercept (level)			Change over time (slope)		
	Estimate	SE	*p*	Estimate	SE	*p*
SBP (Mean)	114.4	0.23	<0.001	2.05	0.07	<0.001
SBP (Variance)	25.5	4.87	<0.001	7.24	2.17	<0.001
DBP (Mean)	66.6	0.16	<0.001	0.82	0.10	<0.001
DBP (Variance)	10.9	2.45	<0.001	0.39	0.28	0.152

### Factors explaining blood pressure growth

**Fig. 3:**
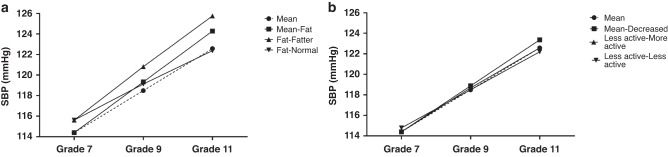
Estimated effects of difference in baseline and change in waist circumference and physical activity on SBP. Example plots showing the estimated effect of differences in baseline and the effect of change in **a** waist circumference (WC) and **b** physical activity (PA) on SBP. These hypothetical scenarios show that children who had increased central adiposity in Grade 7 (increased WC of 5 cm compared to the mean) and children who increased their central adiposity (increased WC by 5 cm over the 4-year measurement period) had higher SBP in Grade 11 than children who maintained the mean WC or children who had increased central adiposity at baseline and reduced their central adiposity (decreased WC of 5 cm over the 4-year measurement period). Similar trends were seen for PA, with less active children or children who reduced their PA over the 4-year measurement period having higher SBP at Grade 11.

Unstandardised (*β*) and standardised regression coefficients (Standardised *β*) for association between baseline measure and changes across a 4-year period on BP trajectories are shown in Table 3. Example plots for the effects of baseline and changes in WC and PA on SBP are shown in Fig. 3.

**Table 3:** Regression coefficients as predictors of intercept and slope of for the development of blood pressure in high school students over 4 years Grades 7–11.

	Intercept				Slope			
	*β*	(SE)	Standardised *β*	*p*	*β*	(SE)	Standardised *β*	*p*
SPB								
Sex (female)	0.803	0.490	0.086	0.101	−2.355	0.388	−0.425	<0.001
Deprivation (IMD)	−0.027	0.033	−0.042	0.409	0.014	0.026	0.038	0.573
Waist circumference (cm)	0.229	0.030	0.395	<0.001	0.069	0.032	0.201	0.031
Physical activity	−0.163	0.492	−0.017	0.740	0.072	0.463	0.013	0.876
Δ Waist circumference (cm)					0.084	0.027	0.256	0.002
Δ Physical activity					0.033	0.325	0.007	0.920
DBP								
Sex (female)	−1.090	0.362	−0.740	<0.001	−0.264	0.294	0.012	0.103
Deprivation (IMD)	0.123	0.022	0.361	<0.001	0.034	0.019	0.124	0.067
Waist circumference (cm)	–0.223	0.349	−0.041	0.523	−0.041	0.025	0.120	0.072
Physical activity	−0.032	0.024	−0.086	0.176	−0.822	0.327	0.242	0.020
Δ Waist circumference (cm)					0.010	0.024	0.004	0.648
Δ Physical activity					−0.650	0.277	0.246	0.019

### Systolic blood pressure

Girls had a lower SBP at baseline and had a smaller increase in SBP over the 4-year measurement period than boys (*β* = −2.355, *p* < 0.001). Higher WC at baseline (Y0) (Grade 7) predicted a higher SBP at baseline (*β* = 0.229, *p* < 0.001) and steeper gradient of the SBP growth trajectory over the 4-year measurement period (*β* = 0.069, *p* = 0.031). An increase in WC over the 4-year period predicted a greater increase in SBP (*β* = 0.084, *p* = 0.002).

There were no significant association between baseline PAQ-A score and baseline (*p* = 0.740) or 4-year change in SBP (*p* = 0.876), or between changes in PAQ-A score over the 4-year period and the gradient of the SBP growth trajectory (*p* = 0.920).

Level of deprivation had no significant effect on baseline (*p* = 0.409) or 4-year growth trajectory (*p* = 0.573) of SBP.

Overall, the strongest influences across the 4-year change in SBP were from baseline and change in WC.

### Diastolic blood pressure

Girls had a significantly lower DBP at baseline (*β* = −1.090, *p* < 0.001) but the smaller increase in DBP over the 4-year measurement period than boys was not significant (*β* = −0.264, *p* = 0.103). Baseline WC was not significantly associated with baseline DBP (*p* = 0.523) or the gradient of the DBP growth trajectory over the 4-year period (*p* = 0.072). There was no significant effect of change in WC over the 4-year measurement period on DBP (*p* = 0.648). Both baseline PAQ-A score (*β* = −0.822, *p* = 0.020) and changes in PAQ-A score (*β* = −0.650, *p* = 0.019) were associated with smaller increases in DBP over the 4-year period. However, there was no relationship between PAQ-A score at baseline and DBP at baseline (*p* = 0.176).

Level of deprivation had a significant effect on baseline (*β* = 0.123, *p* < 0.001) but not 4-year change in DBP (*p* = 0.067).

## Discussion

Hypertension is arguably the most important contributor to CVD, and while it presents in adulthood, hypertension can develop during adolescence.^24^ Since the 1970s, it has been recognised that stratification of BP begins in childhood,^25^ but little research has subsequently considered contributing factors. Elevated BP during childhood tends to track into adulthood,^3^ therefore determining factors associated with the development of elevated BP in youths is necessary to prevent or reduce the disease burden resulting from adult hypertension. This study was carried out in 1501 UK high-school scholars across 4-years (Grades 7–11; between ages 11 and 16) to evaluate the associations between changes in WC, PA, and BP growth trajectory in the EoEHHS. Results showed that WC was significantly associated with SBP, while PA was significantly associated with DBP. These findings suggest that targeted interventions to increase PA and reduce adiposity in adolescents may be required to ameliorate the consistent increases in BP and reduce the risk of future adult hypertension. A novel aspect of this longitudinal study was the use of LGM to identify not only baseline factors that impact development of BP, but the influence of changes in these factors on BP trajectories.

Consistent with previous research showing that blood pressure rises with age,^2^ this study demonstrated an average increase in SBP and DBP over four years but there was considerable variation between individual children. In some children, SBP and DBP increased in a near linear manner whereas others stagnated, increased then decreased or decreased then increased. At baseline, a higher proportion of children had hypertensive SBP (7.2% of boys, 5.9% of girls) and DBP measures (15.3% of boys, 19.3% of girls) compared to reference values,^2^ which was likely related to the high levels of obesity (19.1% of boys, 21.7% of girls) in this study. The reference data were compiled from UK heath surveys conducted in the 1990s and childhood obesity in the UK has since increased. The levels of obesity observed at Grade 7 in this study were comparable to the levels recently reported for UK Grade 6 students (23.4%) and reflect the growing public health problem of adolescent obesity.^1^

There was a positive association between WC at baseline and SBP (Table 3), consistent with previous research showing an association between central adiposity and BP in children.^26–28^ There was also a positive association between baseline WC and growth trajectory of SBP. Children with a higher WC at baseline therefore maintained a higher BP over the 4-year measurement period compared with children who had a lower WC at baseline, and the extent of the difference increased with time. An increase in WC over the 4-year measurement period also predicted a greater increase in SBP (Fig. 3). Baseline or change in WC did not, however, affect baseline or slope of the 4-year growth trajectory for DBP. The higher prevalence of abnormal BP among obese adolescents compared to normal weight adolescents has been confirmed in several studies on different cohorts and populations.^29,30^ Adiposity in adolescence tends to track to adulthood^31^ and a greater BMI during adolescence has been shown to predict adult hypertension.^32^ Adolescent adiposity has also been shown to increase the risk of adult CVD, stroke, and heart failure.^32^ With regards to an increased BP, a higher adolescent BMI has been associated with adult subclinical CVD outcomes such as increased arterial stiffness, greater carotid intima-media thickness (cIMT) and coronary artery calcification.^33^ It has also been demonstrated that the prevalence of elevated BP (≥90 percentile) increases fourfold when BMI exceeds the 85^th^ percentile.^34^ However, an increased central adiposity, rather than subcutaneous adiposity, is thought to more robustly associated with incident hypertension^35^ and a high level of central fatness is a risk factor for elevated BP in adolescents.^36^ This finding is an important consideration in utilising WC rather than body weight or even BMI as predictor measurements for changes in BP, especially in adolescents. While weight gain is expected as children grow, changes in body weight are a function of changes in lean body mass as well as fat mass, and show opposing relations with health risk.^37,38^ Additionally, in children of high-income countries, changes in body weight, are more strongly related to changes in lean body mass than they are adiposity.^39^ Concurrent with the findings of previous studies investigating the effects of changes in adiposity during adolescent,^32,33^ we found a positive association between changes in WC and SBP over the 4-year period, which was of similar magnitude to the effect of baseline WC on baseline SBP. A child with high central adiposity at Year 7 and who increased their central adiposity over the 4-year period (i.e. a fat child who gets fatter) will therefore have the highest SBP at Year 11 (Fig. 3).

Baseline and increases in PAQ-A score over the 4-year period resulted in lower DBP at Grade 11 but did not affect baseline or growth trajectory of SBP. These finding were consistent with previous research reporting the effect of PA on DBP but not SBP.^10,40^ Thus, adolescents with lower PAQ-A scores at baseline had higher DBP 4 years later compared with more active adolescents at baseline. Adolescents who increased their PAQ-A score over the 4-year period had lower DBP at Grade 11. Since LGM’s control for other variables, this effect of PA is independent of central adiposity which generally has been shown to have an inverse relationship with PA in previous studies.^41^ To our knowledge, no previous research has investigated the impact of changes in PA on BP growth trajectories. Increasing PA through various interventions have been shown to improve both SBP and DBP in children aged between 6 and 12 years.^42^ Our results show that adolescents who increase their PA exhibit lower DBP than children who maintain or decrease PA, although the effect of PA on SBP was not significant. Exercise improves arterial compliance, reduces sympathetic tone and can augment endothelium-dependent vasodilation by increased production of nitric oxide which may be a reason for these findings.^43,44^ Although precise mechanisms have yet to be fully elucidated, available data have provided enough information to establish biologically plausible mechanisms for the relationship between PA and hypertension.^12^ It is worth noting that PA behaviours developed during childhood and adolescence have been shown to track to adulthood with moderate to high stability.^45^ Interventions targeting increases in PA are therefore important and can mitigate at least some of the effect of poor baseline values. It has been estimated that as little as a 2 mmHg reduction in population average SBP in adults would reduce coronary heart disease mortality by 4%, while a reduction of 5 mmHg would reduce coronary heart disease mortality by 9%.^46^ Further, it is estimated that a 2 mmHg reduction in the population average DBP would result in a 17% decrease in the prevalence of hypertension.^47^ Previous research has shown that PAQ-A score declines by an average of 0.5 points between Grades 7 and 11.^19^ Adolescents with a 0.5-point higher PAQ-A score at baseline would experience a 1.64 mmHg smaller increase in DBP over the four-year period (0.41 mmHg each year), while adolescents who increased their PAQ-A score by 0.5 points over the study period would experience a 1.3 mmHg smaller increase in DBP over the 4-year period (0.33 mmHg each year: Table 3). The PAQ-A score is a mean of Likert scale responses; therefore, it is not possible to directly relate these results to objective PA recommendations. Broadly, increasing PAQ-A score by 1-point translates to performing activities an additional 1–2 times a week and moving from ‘hardly ever’ to ‘sometimes’ or ‘sometimes’ to ‘quite often’ across most activities. While these changes do not translate directly to reductions in DBP as this increases with growth, maintaining or increasing PA during adolescence could slow the rise in DBP over time. This is meaningful for future hypertension and CVD risk reduction into adulthood.

While previous cross-sectional research has reported that PA is negatively associated with BP in adolescents,^9–11^ in this and a previous study,^48^ baseline measures of PA were not associated with baseline measures of SBP or DBP. Some of this disparity can be explained by methodological differences in studies such as the use of objective or self-reported measures of PA, and the association between adiposity and both PA and BP which might confound results. In addition, the PAQ-A only measures overall PA and does not differentiate between light, moderate and vigorous intensity activity which might have contributed to the inconsistencies in findings. There is evidence to suggest that vigorous PA is more beneficial than moderate PA for a variety of health outcomes in adults,^49,50^ and UK guidelines include specific recommendations for vigorous PA in individuals aged 19–64 years.^51^ While there are no such recommendations for children, the benefits of vigorous PA on cardiometabolic risk factors are becoming increasingly recognised.^52–55^

Our findings have important clinical and public health implications for the prevention of CVD, especially those related to high BP. Children with elevated BP who are able to normalise their BP in the period between childhood and adulthood reduce the likelihood of future CVD compared to those who normalise their BP in adulthood, highlighting the importance of identifying and managing factors that contribute to elevated BP in childhood.^56^ Due to absence of data to link a specific BP level in childhood with adverse outcomes in adulthood, hypertension in childhood has been defined statistically as a BP level that is consistently ≥95th percentile of the normative BP distribution, adjusted for age, sex, and height. The goals of treatment and management are to reduce SBP and DBP <90th percentile.^57^

### Limitations

This study has several limitations. Blood pressure measures are a product of initial values and change over time and the intercept can only be predicted by past factors (e.g. how active an individual has been, resulting in how fit they are etc.). Using regression models to predict 4-year changes using only baseline values is problematic, because these are affected by the law of initial values and regression to the mean.^58^ This means that if an individual has a high baseline BP, it is likely to increase by a lesser amount than someone who has a low baseline BP, because populations homogenise over time. However, the longitudinal design and use of LGM allowed for the estimation of inter-individual variability in intra-individual trajectories of change over time.^59^ Further, the use of LGM accounts for missing values, whereas participants with missing values are excluded when using General Linear Models.

Since BP measures were taken at a single visit, the ‘white coat affect’ likely influenced these. It has been estimated that up to half of children referred for evaluation of elevated BP have white coat hypertension.^60^ The use of ambulatory BP monitoring should therefore be considered in future studies. Further, PAQ-A score was self-reported. Using self-report assessments is convenient in large samples but the results can be inconsistent,^61^ and inconsistency might have been magnified in this study because cognitive ability and comprehension of children changes significantly between Grades 7 and 11. This might have introduced random measurement error which can attenuate associations and thus induce bias.^62^ Additionally, WC was used as a measure of central adiposity and while this measure has been shown to be valid, it is nonetheless an indirect measure and more advanced direct measures (e.g. dual-energy X-ray absorptiometry) would provide better estimates of overall adiposity. However, such measures are impractical in large-scale studies. Finally, because there were no data collected for nutritional habits, smoking drinking habits or parental cardiovascular disease, it was not possible to include these in our models. At the time of design, it was decided that assessment of nutritional status (in addition to the anthropometric, physiological, and PA information captured at three time points) would place an unacceptable burden on participants and schools. Our participants were all under the age of 18 years (the legal age to smoke and drink in the UK) therefore these behaviours are not representative^63–65^ and any self-report data were likely to be biased. Parental ages predominantly ranged from 40 to 50 years, so a high prevalence of cardiovascular disease was unlikely.

## Conclusion

To conclude, adolescents who increased their WC relative to the mean had significantly higher SBP at Grade 11. Baseline and changes in PAQ-A score during adolescence were negatively associated with the 4-year growth trajectory of DBP. Specifically, adolescents with low PA at baseline, and/or adolescents who decreased their PA over the 4-year period exhibited the highest DBP at Grade 11. Regardless of baseline values, interventions that increase PA and reduce adiposity in adolescents could result in meaningful reductions in BP at Grade 11, which, in turn, could reduce risk of hypertension and manifest CVD into adulthood. Our findings underscore the prognostic value of PA and WC in predicting changes in blood pressure in adolescents. As such, these results could be valuable in planning related age-specific programmes to prevent hypertension in similar cohorts in other high-income countries and prevent later adult hypertension.

### Supplementary information

Supplementary Information

## Data Availability

Data are available on the UK Data Archive: 10.5255/UKDA-SN-7456-1.
