# Supplementary material for: Four-year longitudinal associations of physical activity, waist circumference, and blood pressure in UK adolescents
Source: Pediatr Res. 2023 Oct 13;95(3):736–43. doi: 10.1038/s41390-023-02837-2 (PMC10899106; doi:10.1038/s41390-023-02837-2)
Supplement: Supplementary file 1 — Supplementary Information [file 41390_2023_2837_MOESM1_ESM.pdf]

Supplementary Information 1

The following provides the model fit parameters for a) systolic blood pressure and b) diastolic blood pressure latent growth models.

a) **Systolic Blood Pressure**

Model fit Systolic Blood Pressure: Chi-square Test

**Model**	χ^2	**df**	**p**
Baseline model	159.903	3	
Growth curve model	3.316	1	0.069

Parameter estimates: Latent curve

**Component**	**Parameter**	**Estimate**	**SE**	**z-value**	**p**	**95%**	**CI**
Intercept	Mean	114.45	0.231	495.96	<.001	114	114.9
	Variance	25.467	4.87	5.23	<.001	15.92	35.01
Linear slope	Mean	4.091	0.146	28.10	<.001	3.81	4.34
	Variance	7.241	2.171	3.34	<.001	2.99	11.49

**Latent covariances**			**Estimate**	**SE**	**z-value**	**p**	**95%**	**CI**
Intercept	↔	Linear slope	-7.145	2.729	-2.618	0.009	-12.49	[illegible].80

Residual variances

**Variable**	**Estimate**	**SE**	**z-value**	**p**	**95%**	**CI**
SBP 1	53.52	5.19	10.30	< .001	43.34	63.71
SPB 2	76.65	3.37	22.70	< .001	70.04	83.27
SBP 3	32.99	4.31	7.65	< .001	24.53	41.44

The Latent covariance between intercept and slope was -7.14 (2.73) mmHg which was statistically significant (p=0.009) indicating higher initial SBP (intercept) values were associated with a smaller increase (slope) over time.

Additional Fit measures

**Index**	**Value**
Comparative Fit Index (CFI)	0.999
Tucker-Lewis Index (TLI)	0.999
Bentler-Bonett Non-normed Fit Index (NNFI)	0.999
Bentler-Bonett Normed Fit Index (NFI)	0.974
Parsimony Normed Fit Index (PNFI)	0.371
Bollen's Relative Fit Index (RFI)	0.932
Bollen's Incremental Fit Index (IFI)	1.000
Relative Noncentrality Index (RNI)	1.000

**Other fit measures**	**Value**

Root mean square error of approximation (RMSEA)	0.004
RMSEA 90% CI lower bound	0.000
RMSEA 90% CI upper bound	0.033
RMSEA p-value	0.999
Standardized root mean square residual (SRMR)	0.010
Hoelter's critical N (α = .05)	2485.8
Hoelter's critical N (α = .01)	3218.9
Goodness of fit index (GFI)	1.000
McDonald fit index (MFI)	1.000
Expected cross validation index (ECVI)	0.035

Results showed that the systolic blood pressure model fitted the data well. The $\chi 2$-test was not significant despite the large sample size. The CFI and TLI were > 0.95, the RMSEA was < 0.070 and the SRMR was <0.08. The GFI (proposed as a more stringent and appropriate measure[1]) was <0.95.

The model for SBP also passed Hu and Bentler's combination rules for RMSEA <0.06 and SRMR <0.09; and combination rules for CFI >0.96 and an SRMR <0.09.[2]

a) **Diastolic Blood Pressure**

Model fit - Diastolic Blood Pressure: Chi-square Test

Model	χ^2	df	p
Baseline model	101.397	3	
Growth curve model	2.487	1	0.115

Parameter estimates Latent curve

Component	Parameter	Estimate	SE	z-value	p	95%	CI
Intercept	Mean	66.61	0.17	396.85	< .001	66.28	66.94
	Variance	10.94	2.45	4.46	< .001	6.13	15.74
Linear slope	Mean	1.63	0.10	15.62	< .001	1.43	1.83
	Variance	1.58	0.10	4.13	0.002	0.58	3.74

Latent covariances			Estimate	SE	z-value	p	95%	CI
Intercept	↔	Linear slope	-2.89	1.41	-2.06	.040	-5.65	-1.02

Residual variances

Variable	Estimate	SE	z-value	p	95%	CI
DBP 1	33.19	2.73	12.15	< .001	27.84	38.55
DBP 2	34.65	1.55	22.40	< .001	31.62	37.69
DBP 3	20.79	2.06	10.09	< .001	16.75	24.83

The Latent covariances between intercept and slope of -2.89 (1.41) mmHg was statistically significant (p=0.04) indicating higher initial SBP (intercept) values were associated with smaller increases (slope) over time.

Additional Fit indices

Index	Value
Comparative Fit Index (CFI)	0.995
Tucker-Lewis Index (TLI)	0.987
Bentler-Bonett Non-normed Fit Index (NNFI)	0.987
Bentler-Bonett Normed Fit Index (NFI)	0.959
Parsimony Normed Fit Index (PNFI)	0.365
Bollen's Relative Fit Index (RFI)	0.891
Bollen's Incremental Fit Index (IFI)	0.995
Relative Noncentrality Index (RNI)	0.995

Other fit measures

Metric	Value
Root mean square error of approximation (RMSEA)	0.010
RMSEA 90% CI lower bound	0.000
RMSEA 90% CI upper bound	0.035
RMSEA p-value	0.999
Standardized root mean square residual (SRMR)	0.013
Hoelter's critical N (α = .05)	2248.1
Hoelter's critical N (α = .01)	2913.8
Goodness of fit index (GFI)	1.000
McDonald fit index (MFI)	1.000
Expected cross validation index (ECVI)	0.036

Results showed that the diastolic blood pressure model fitted the data well. The χ^2-test was not significant despite the large sample size. The CFI and TLI were > 0.95, the RMSEA was < 0.070 and the SRMR was <0.08. The GFI (proposed as a more stringent and appropriate measure[1]) was also <0.95.

The model for DBP also passed Hu and Bentler’s combination rules for RMSEA <0.06 and SRMR <0.09; and combination rules for CFI >0.96 and an SRMR <0.09.[2]

Supplementary Information 2
The following shows the frequency and patterns of missing data points for all independent and dependent variables across the three measurement points.

Table S2a Univariate Statistics for Missing Values

	N	Mean	SD	Missing (n)	Missing (%)
SBP1	1342	114.6	8.9	80	5.6
SBP2	1349	118.1	9.7	73	5.1
SBP3	1382	122.7	7.7	40	2.8
DBP1	1362	66.7	6.6	60	4.2
DBP2	1346	68.0	6.4	76	5.3
DBP3	1384	69.9	5.1	38	2.7
WC1	1419	65.9	8.1	3	0.2
WC2	1411	69.5	6.9	11	0.8
WC3	1408	71.6	5.4	14	1.0
PA1	1355	2.9	0.5	67	4.7
PA2	1353	2.7	0.5	69	4.9
PA3	1393	2.6	0.5	29	2.0
SES (IMD)	1412	11.7	7.3	10	0.1%

Legend: SBP – Systolic Blood Pressure (mmHg); DBP – Diastolic Blood Pressure (mmHg), WC – Waist circumference (cm) PA- Physical Activity (PAQ-A Score). Time 1 – Grade 7, Time 2 – Grade 9, Time 3 Grade 11.

Table S2b. Cross-tabulated patterns of missing data.
Legend: SBP – Systolic Blood Pressure (mmHg); DBP – Diastolic Blood Pressure (mmHg), WC – Waist

Tabulated Patterns													
N	Missing Patterns[a]												
	WC1	WC2	WC3	PA3	SBP3	DBP3	DBP1	PA2	PA1	DBP2	SBP 2	SBP1	[b]
962													962
22					X								984
56												X	1018
44										X			1006
42								X					1004
48									X				1010
52											X		1014
27						X							989
16				X									978
45							X						1007

circumference (cm) PA- Physical Activity (PAQ-A Score). Time 1 – Grade 7, Time 2 – Grade 9, Time 3 Grade 11.
Patterns with less than 1% cases (14 or fewer) are not displayed
a Variables are sorted on missing patterns
b Number of complete cases if variables missing in that pattern (marked with X) are not used

Table S2c. Summary of Actual and Means and Standard Deviations and Estimates Derived from Expectation-Maximisation.

	Values (Mean)	Values (SD)	EM (Mean)	EM (SD)
SBP1	114.56	8.89	114.64	8.92
SBP2	118.12	9.74	118.22	9.76
SBP3	122.73	7.68	122.70	7.69
DBP1	66.71	6.65	66.73	6.65
DBP2	68.01	6.43	68.03	6.44
DBP3	69.94	5.15	69.95	5.15
WC1	65.90	8.11	65.88	8.11
WC2	69.54	6.93	69.51	6.93
WC3	71.60	5.43	71.59	5.43
PA1	2.85	0.49	2.85	0.49
PA2	2.66	0.53	2.66	0.53
PA3	2.60	0.46	2.60	0.46

Legend: EM – Expectation-Maximization. SBP – Systolic Blood Pressure (mmHg); DBP – Diastolic Blood Pressure (mmHg), WC – Waist circumference (cm) PA- Physical Activity (PAQ-A Score). Time 1 – Grade 7, Time 2 – Grade 9, Time 3 Grade 11.

a-Note that expectation-maximization was not used to impute missing values. The values shown here are illustrative only. The close approximation of means and SD values obtained using EM and values computed from complete cases only illustrates how non-biased estimates can be derived when data are missing completely at random. (*Little's MCAR test: Chi-Square = 627.3, DF = 572, Sig = 0.054)*

Complete data were available for 73.1% (n=1039) of participants at all three measurement points. Overall, there were n=473 missing values for BP, WC, and physical activity (PA) – equivalent to 2.7% of all values potentially available across the three measurement points.
Little's MCAR test was not significant ($\chi2$=627.3, df=572, p=.054) indicating that values were missing completely at random. Importantly, there were no meaningful differences in measures of BP, WC, or PA (all d<0.2). Based on this evidence, data were confirmed as missing completely at random (missingness could not be accounted for by the values of variables themselves nor explained by the values of other variables included in the analysis).
